# Etiology, Clinical, Radiological, and Microbiological Profile of Patients with Non-cystic Fibrosis Bronchiectasis at a Tertiary Care Hospital of Pakistan

**DOI:** 10.7759/cureus.7208

**Published:** 2020-03-08

**Authors:** Nadia Sharif, Mirza Saifullah Baig, Sana Sharif, Muhammad Irfan

**Affiliations:** 1 Pulmonology, Dow University of Health Sciences, Karachi, PAK; 2 Epidemiology and Public Health, University of Saskatchewan School of Public Health, Saskatoon, CAN; 3 Medicine, The Aga Khan University, Karachi, PAK

**Keywords:** etiology, microbiology, complications, non cf bronchiectasis

## Abstract

Objectives

To identify the etiology of non-cystic fibrosis bronchiectasis (NCFB), to assess the clinical presentation, radiological findings, and microbiological profile of patients presenting with a diagnosis of bronchiectasis in a tertiary care center of Pakistan.

Methods

This was a prospective observational cohort study where patients with a diagnosis of bronchiectasis proven by high-resolution computed tomography (HRCT) were evaluated for etiology, clinical characteristics, microbiology, radiology, spirometric profile, and in-hospital outcomes.

Results

During the study period, 196 patients were diagnosed with NCFB. The majority of the patients were men 76.5% (n = 150) and 83.6% (n = 163) of the total patients were younger than 60 years of age. The majority of these patients (58.7%, n = 111) had a duration of symptoms between 5-10 years. The etiology of bronchiectasis was identified in 92.9% of cases. Post-infectious bronchiectasis was the most common cause (67.8%, n = 133), followed by chronic obstructive pulmonary disease (COPD) (9.2%, n = 18), and allergic bronchopulmonary aspergillosis (ABPA) (7.1%, n = 14). Among the post infectious causes, a history of TB was present in 85% (n = 114/133) of patients. Obstructive impairment was the most common spirometric pattern, observed in 68.9% (n = 135) of patients. *Pseudomonas aeruginosa* was the most commonly isolated organism (36.2%, n = 71). Hemoptysis was the most frequent complication found in 20.9% of patients (n = 41). Out of these 196 patients, 94.4% (n = 185) received medical management and were discharged from the hospital. Respiratory failure was significantly associated with the Pseudomonas group as compared to non-pseudomonas group [(n = 21 (29%) vs n = 18 (14.4%) p = 0.01]. During hospitalization seven patients (3.6%) were died because of respiratory failure.

Conclusions

Post TB bronchiectasis was the leading cause of non-cystic fibrosis (CF) bronchiectasis in this cohort, with Pseudomonas was the commonest pathogen isolated from the respiratory specimen, which was significantly associated with respiratory failure. On spirometry, obstructive impairment was found in the majority of patients and hemoptysis was the most frequent complication.

## Introduction

Bronchiectasis is a chronic respiratory condition that was first described by Laënnec in 1819 [1]. High-resolution computed tomography (HRCT) is considered the gold standard for its diagnosis [2,3]. There are various clinical conditions, which can lead to this chronic debilitating condition: post-infectious, allergic bronchopulmonary aspergillosis (ABPA), immunodeficiency syndromes, connective tissue diseases (CTD), cystic fibrosis (CF), chronic respiratory conditions like asthma, chronic obstructive pulmonary disease (COPD), primary ciliary dyskinesia (PCD) and cases with unknown etiology, i.e., idiopathic [4,5]. Since these conditions differ in their management and prognosis, diagnosis of underlying etiology is important.

Although CF-associated bronchiectasis has been studied extensively, non-cystic fibrosis bronchiectasis (NCFB) since long considered an orphan lung disease [6]. Recently there has been a growing interest in this rather neglected disease since it is the cause of significant morbidity and mortality all over the world [4].

There is a geographic variation in clinical features and etiologies of bronchiectasis [7]. In earlier studies, the cause of bronchiectasis remained elusive in the majority of patients, however, immune dysregulation was found to be the leading cause of bronchiectasis in a study from the United States (US) [8]. On the contrary, post-infectious bronchiectasis was the predominant underlying etiology in a recently published data from India and a study from China [9,10].

As described by Cole’s “vicious cycle model, bronchiectasis is associated with microbiological colonization of airways with recurrent infections and chronic inflammation leading to impaired mucociliary clearance and progressive lung damage [11]. So infections play a major role in the progression of bronchiectasis, resulting in significant morbidity and mortality. Although *Haemophilus influenzae, Pseudomonas aeruginosa, Moraxella catarrhalis, Staphylococcus aureus,* and Enterobacteriaceae are commonly isolated pathogens from bronchiectatic airways, microbiological colonization with some organisms has proven to have worse outcomes than others [12]. From our knowledge in CF patients, we know colonization, with Pseudomonas, is associated with a decline in lung function with frequent exacerbations and mortality [13]. This is because of its tendency to form biofilms and the ability to develop antibiotic resistance. Similar results were shown in data from 21 observational studies including patients with both CF and NCFB [14]. The US bronchiectasis registry showed that nontuberculous mycobacteria (NTM) were more commonly found in women, with bronchiectasis diagnosed at a later age and gastroesophageal reflux disease (GERD) as the predominant underlying condition [15]. A study by Faverio et al. showed the patients with bronchiectasis who grew NTM in respiratory samples had a milder disease and better pulmonary functions when compared to patients who had Pseudomonas [16]. So outcomes of bronchiectasis are also dependant on the isolated microorganism.

Because of the heterogeneous nature of this chronic debilitating condition, with evidence of geographic variation in etiologies and other clinical features and since sparse data is available from developing countries especially from South East Asia, we found it imperative to collect our data. The aim of the study was to identify the underlying etiology of bronchiectasis, to assess the clinical presentation, radiological findings, and microbiological profile of patients who presented to our centre with a suspected diagnosis of bronchiectasis.

## Materials and methods

Methods

This was a prospective observational cohort study that was conducted in the inpatient department of Ojha Institute of Chest Diseases from March 2017 till May 2019. Our institute is a 180 bedded tertiary care center for adult respiratory diseases including Tuberculosis (TB). HRCT was done for all patients with suspicion of bronchiectasis based on history, clinical exam, and chest X-ray findings. All patients who were diagnosed to have bronchiectasis based on HRCT findings, who gave written informed consent to be the part of the study were included and a study proforma was filled for them. Patients who were known to have CF or those who were found to have CF during the evaluation were excluded from the study. Patients with interstitial lung disease (ILD) and traction bronchiectasis were excluded.

In history, the emphasis was laid on previous severe lower respiratory tract infections, pneumonia, TB, asthma, COPD, smoking status, history of inflammatory bowel disease, history of GERD or neurological conditions predisposing to chronic aspiration, signs, and symptoms suggestive of CTD, and duration of symptoms. The age was divided into four groups, i.e., age between 15-30 years, 31-45 years, 46-60 years, and >60 years. The duration of symptoms was also divided into three groups, i.e., between 1-5 years, 5-10 years, and >10 years. Workup to evaluate the diagnosis of bronchiectasis was done as per British Thoracic Society (BTS) guidelines for bronchiectasis [5].

Patients were tested for immunoglobins levels (IgA, IgM, IgG, IgE) who had a prolonged history of recurrent infections with bilateral extensive bronchiectasis (involving more than two lobes). In case if immunoglobins levels came out to be normal, the patient was screened for CF with two sets of sweat chloride tests and Delta F508 (only available genetic test in Pakistan). Patients with a diagnosis of CF were excluded from the study. Once both hypogammaglobulinemia and CF were excluded, the saccharine test was done in patients with bilateral extensive bronchiectasis. If this test was turned out to be positive, with a history of otitis media, infertility or dextrocardia, the patient was labeled to have PCD. Diagnosis of bronchiectasis secondary to COPD was made in patients with a history of significant smoking, i.e. >15 pack-year, with spirometric diagnosis of post-bronchodilator obstructive airway impairment and evidence of background emphysema, and if symptoms appeared after a history of significant smoking with other pertinent workup inconclusive for any other condition which can lead to bronchiectasis. Diagnosis of post TB or post pneumonia bronchiectasis was made when the suggesting symptoms followed soon after a severe infection. Patients with a history of asthma and evidence of proximal bronchiectasis were evaluated further with Serum IgE, Aspergillus specific IgE, and Aspergillus skin test. These patients were labeled to have underlying ABPA based on established ISHAM criteria for the diagnosis of ABPA [17]. For the patients with a history suggestive of arthritis and CTD, workup including rheumatoid factor, anti-CCP, and antinuclear antibody (ANA) profile were sent. An extended panel for CTD was sent if these results were negative but history did point towards any other connective tissue disorder. Alpha-1 antitrypsin level was sent only in patients with evidence of emphysema along with bronchiectasis. Patients were labeled to have idiopathic bronchiectasis if all of the above-mentioned workups did not point toward a specific diagnosis. Complications of bronchiectasis were noted as well.

Radiological extent of bronchiectasis was divided into unilateral upper lobe (UUL), bilateral upper lobes (BUL), unilateral lower lobe (ULL), bilateral lower lobes (BLL), lingual and middle lobe (ML&L), diffuse unilateral bronchiectasis [DUB (all the lobes of one hemithorax involved)], diffuse bilateral bronchiectasis [DBB (bilaterally if three or more lobes are involved)]. Spirometry was intended to be performed in all patients. The spirometric data were categorized into normal, obstructive and possible restriction/nonspecific as per the ATS/ERS task force statement on standardization of lung function testing [18].

In patients having productive cough, sputum was sent for bacterial, AFB (acid fast bacilli), and fungal cultures. Sputum sample which grew two or more organisms was labeled to have a mixed population. Bronchoscopy was performed in patients who had hemoptysis to localize the site of bleeding, in patients whose sputum culture did not grow any organism and the patient was not clinically improving on empiric treatment or patients with focal bronchiectasis to rule out proximal obstruction. Patients were followed up until the time of discharge to find the outcome of the patients during hospitalization.

Since we know from previous literature, isolation of *Pseudomonas aeruginos*a is associated with worse outcomes in patients with bronchiectasis, we divided the patients into Pseudomonas and non-Pseudomonas group based on the presence or absence of Pseudomonas in sputum in order to find differences in presentation and clinical outcomes between the groups.

Statistical analysis

Data were analyzed using SPSS (Statistical Package for Social Sciences) version 26.0 (IBM Corp., Armonk, NY). Frequencies and percentages were calculated for the etiologies, clinical, radiological, microbiological findings, spirometric data, and complications. Patients were divided into Pseudomonas and non-Pseudomonas based on sputum microbiology. The categorical data of the two groups, i.e., presentation, complications, and hospital outcomes were compared using the Chi-square test. P-value of less than 0.05 was considered significant.

## Results

A total of 202 patients with the diagnosis of bronchiectasis based on HRCT findings were enrolled from March 2017 till May 2019. During evaluation six patients were excluded from the study after being diagnosed to have CF, so 196 were included in the final analysis. In this cohort, 76.5% (n = 150) were men. The age of the patients was divided into four groups: between 15 to 30 years, 31 to 45 years, 45 to 60 years and greater than 60 years. The majority of them (83.2%, n = 163) were younger than 60 years of age. Of these 196 patients, 95.9% (n = 188) were admitted with an exacerbation while rest 4.1% (n = 8) had chronic stable bronchiectasis and admitted with other medical issues. The majority of these 58.2% (n = 111) patients had a duration of symptoms in between 5-10 years, with 29.1% between 1-5 years and 12.8% had chronic symptoms for greater than 10 years. The clinical characteristics of patients are summarized in Table 1.

**Table 1:** Clinical characteristic and outcome of patients

Characteristics		Frequencies n (%)
Age	15 to 30 years	53 (27%)
	31 to 45 years	51 (26%)
	46 to 60 years	59 (30.1%)
	>60 years	33 (16.8%)
Gender	Men	150 (76.5%)
	Women	46 (23.5%)
Duration of symptoms	1 to 5 years	57 (29.1%)
	5 to 10 years	114 (58.2%)
	> than 10 years	25 (12.8%)
Spirometry	Obstructive	135 (68.9%)
	Nonspecific/possible restriction	40 (20.4%)
	Normal	5 (2.6%)
	Couldn’t perform	16 (8.2%)
Signs and Symptoms	Daily cough	191 (97.4%)
	Daily sputum production	165 (84.2%)
	Dyspnea	153 (78.1%)
	Crepts on auscultation	118 (60.2%)
	Fever	90 (45.9%)
	Clubbing	66 (33.7%)
	Wheezing	55 (28.1%)
Clinical presentation	Exacerbation	188 (95.9%)
	Chronic case	8 (4.1%)
In hospital outcome	Medically managed	185 (94.4%)
	Referred to surgery	4 (2%)
	Died	7 (3.6%)

**Figure 1:**
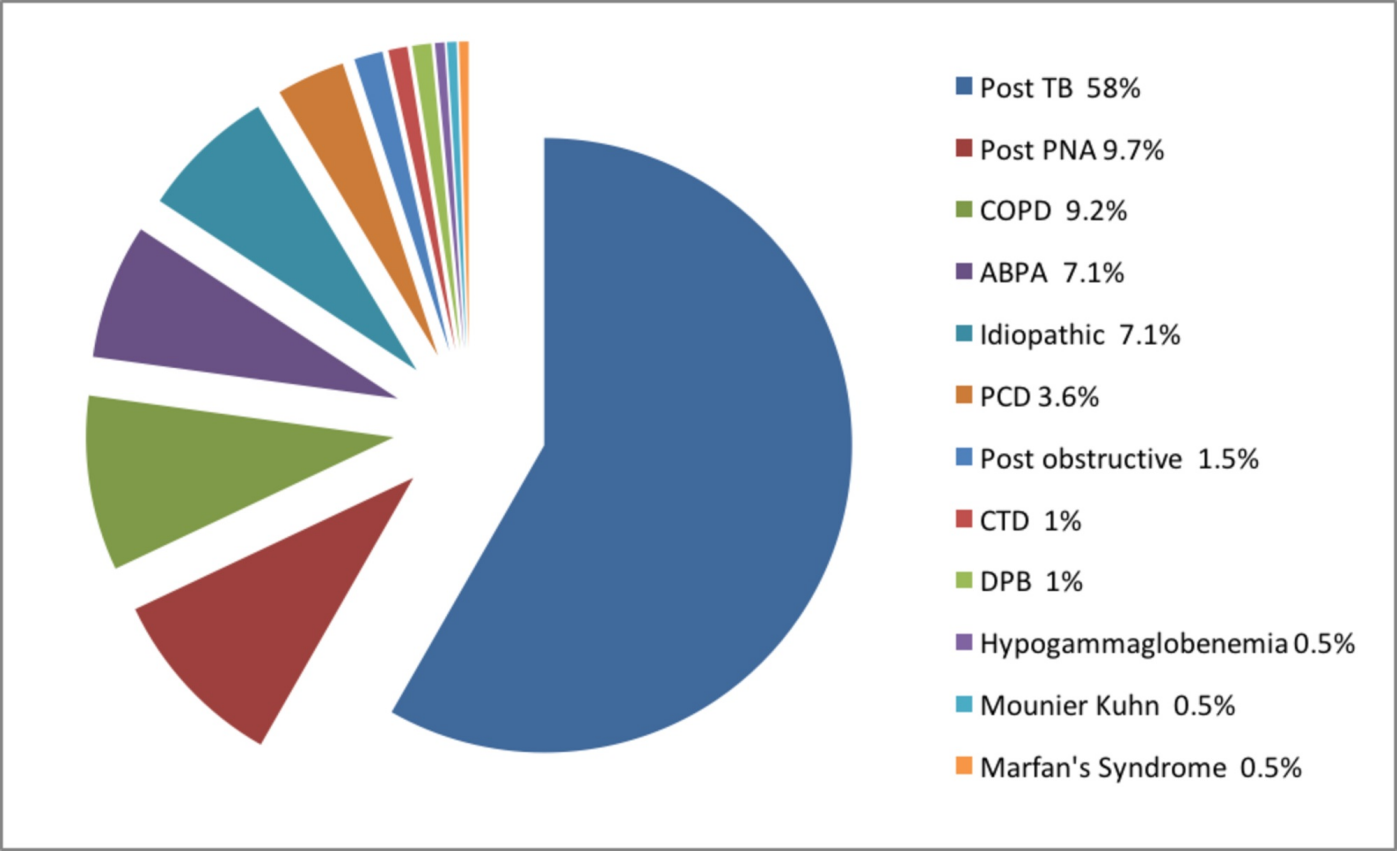
Etiologies of non-cystic fibrosis (CF) bronchiectasis

The etiology of bronchiectasis was identified in 92.9% (n = 182) while in 7.1% (n = 14) cause remained unknown. Figure 1 shows post infectious bronchiectasis was the most common cause (67.8%, n = 133) out of which a history of TB was present in 85% (n = 114/133) and a history of pneumonia in 14% (n = 19/133). It was followed by COPD (9.2%, n = 18), ABPA (7.1%, n = 14), PCD (3.6%, n = 7), diffuse pan bronchiolitis (DPB), rheumatoid arthritis and foreign body inhalation 1% (n = 2) each, post obstructive bronchiectasis (secondary to carcinoid tumor), Marfan syndrome (MFS), Mounier-Kuhn syndrome (MKS) and primary antibody deficiency (PAD) 0.5% (n = 1) each. During study period no patient was diagnosed to have chronic aspiration and inflammatory bowel disease.

Spirometry was normal in only 2.6% (n = 5) cases. Obstructive impairment was observed in 68.9% (n = 135) and 20.4% (n = 40) had nonspecific impairment. Due to advanced age or severity of symptoms, 16 patients (8.2%) were sent for spirometry but they were unable to perform the spirometry.

**Figure 2:**
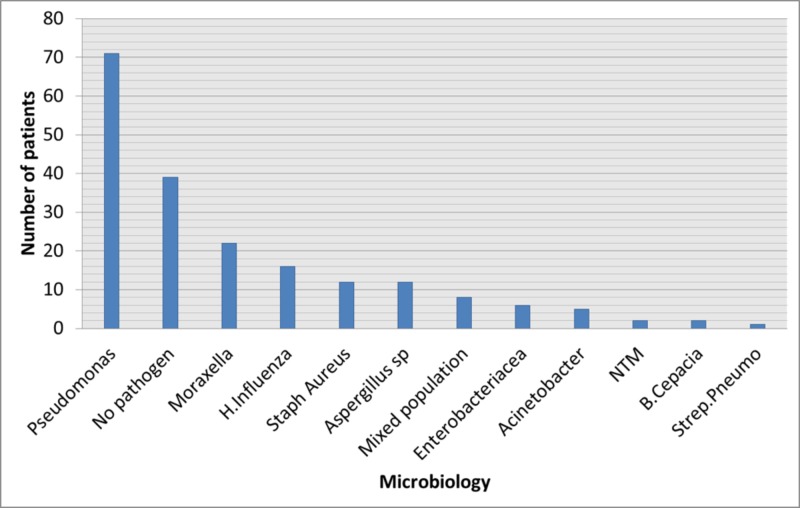
Etiologies of non-cystic fibrosis (CF) bronchiectasis

*Pseudomonas aeruginosa* was the most common organism (36.2%, n = 71) identified on sputum culture. In 39 patients (19.9%) either no pathogen was isolated or had normal commensals. The other significant isolated organisms were: *Moraxella catarrhalis *(11.2%, n = 22), *Haemophilus influenza* (8.2%, n = 16), Aspergillus species and *Staphylococcus aureus* 6.1% (n = 12) each, mixed population (4.1%, n = 8), *Enterobacteriaceae* (3.1%, n = 6), *Acinetobacter* (2.6%, n = 5), NTM and *Burkholderia cepacia* 1% (n = 2) each and *Streptococcus pneumonia *(0.5%, n = 1). Figure 2 shows frequency of isolated pathogens.

Radiologically, upper lobe predominant disease pattern was found in majority of patients (41.8%, n = 82) with 24% (n = 47) had bilateral upper lobe involvement (BUL) and 17.9% (n = 35) had unilateral upper lobe (UUL) bronchiectasis. The other radiological distributions were: Diffuse bilateral bronchiectasis (DBB) found in 20.4% (n = 40), diffuse unilateral bronchiectasis (DUB) in 15.8% (n = 31), bilateral lower lobe (BLL) in 12.2% (n = 24), unilateral lower lobe (ULL) in 8.2% (n = 16), both middle lobe and lingula involvement (ML&L) in 1% (n = 2) and isolated middle lobe (ML) in 0.5% (n = 1).

These patients were also assessed for any associated complications. Hemoptysis was the most frequent complication found in 20.9% patients (n = 41). It was followed by respiratory failure requiring supplemental oxygen/NIV (19.9%, n = 39), pneumonia (4.6%, n = 9), cor pulmonale (4.1%, n = 8), lung abscess and pneumothorax 1.5% (n = 3) each, empyema and secondary amyloidosis in 0.5% (n = 1) each. In nearly half of the patients (46.4%, n = 91) no complication was identified.

Out of these 196 patients, 94.4% (n = 185) were managed medically and discharged from the hospital. Four patients with focal bronchiectasis, with history of recurrent symptoms and hemoptysis, were referred to thoracic surgeons for lobectomy. Five of the patients with massive hemoptysis required bronchial artery embolization. Because of respiratory failure seven patients (3.6%) died during hospitalization.

Table 2 summarises the comparison between Pseudomonas and non-Pseudomonas group and Table 3 compares the characteristics of patients among different etiologies.

**Table 2:** Comparison of characteristics of Pseudomonas and non-Pseudomonas group

N (%)	Pseudomonas n = 71 (%)	Non-Pseudomonas n = 125 (%)	p-value
Presentation			0.26
Chronic case	1 (1.4%)	7 (6%)	
Exacerbation	70 (98%)	118 (94%)	
Complications			
Pneumonia	1 (1.4%)	8 (6.4%)	0.16
Empyema	0 (0)	1 (0.8%)	1.00
Lung Abscess	0 (0)	3 (2.4%)	0.55
Respiratory failure	21 (29%)	18 (14.4%)	0.01*
Cor pulmonale	5 (7%)	3 (2.4%)	0.14
Pneumothorax	2 (3%)	1 (0.8%)	0.29
Hemoptysis	13 (18%)	28 (22.4%)	0.58
None	29 (41%)	63 (50.4%)	0.23
Duration of symptoms			0.26
1 to <5 years	16 (22.5%)	41 (33%)	
5 to 10 years	47 (66%)	67 (57%)	
>10 years	8 (11%)	17 (14%)	
In hospital outcome			0.28
Medically management	69 (97%)	116 (93%)	
Referred to surgeon	0 (0)	4 (3%)	
Died	2 (3%)	5 (4%)	

**Table 3:** Comparison of characteristics of different etiologies Post PNA: Post pneumonia; COPD: Chronic obstructive pulmonary disease; ABPA: Allergic bronchopulmonary aspergillosis; PCD: Primary ciliary dyskinesia; Post obs: Post obstructive; CTD: Connective tissue disease; DPB: Diffuse pan bronchiolitis; PAD: Primary antibody deficiency; MKS: Mounier Kuhn syndrome; MFS: Marfan syndrome; UUL: Unilateral upper lobe; BUL: Bilateral upper lobe; DBB: Diffuse bilateral bronchiectasis; DUB: Diffuse unilateral bronchiectasis; BLL: Bilateral lower lobe; ULL: Unilateral lower lobe; ML&L: Middle lobe & lingual; ML: Middle lobe.

	Post TB n = 114	Post PNA n = 19	COPD n = 18	ABPA n = 14	Idiopathic n = 14	PCD n = 7	Post obs n = 3	CTD n = 2	DPB n = 2	PAD n = 1	MKS n = 1	MFS n = 1
Age (y) n (%)												
15-30	19 (17)	10 (53)	0	4 (29)	8 (57)	4 (57)	1	0	1 (50)		0	1 (100)
31-45	29 (25)	3 (16)	0	8 (57)	5 (36)	2 (29)	1	1 (50)	1 (50)	1 (100)	0	0
46-60	44 (38)	2 (10)	10 (56)	2 (14)	1 (7)	1 (14)	1	1 (50)	0	0	0	0
>60	22 (19)	4 (21)	8 (44)	0	0	0	0	0	0	0	1 (100	0
Sex n (%) Male	90 (79)	13 (68)	12 (67)	10 (71)	11 (79)	6 (86)	3 (100)	0	2 (100)	1 (100)	1 (100)	1 (100)
Radiology n (%)												
UUL	32 (28)		0	0	0	0	1 (33)	0	0	0	0	0
BUL	45 (39)	2 (10)	1 (5)	1 (7)	0	0	0	0	0	0	0	0
DBB	6 (5)	0	3 (17)	13 (93)	7 (50)	7 (100)	0	0	2 (100)	1 (100)	1 (100)	0
DUB	28 (25)	1 (5)	0	0	0	0	1 (33)	0	0	0	0	0
BLL	0	2 (10)	13 (72)	0	6 (43)	0	0	2 (100)	0	0	0	0
ULL	3 (3)	3 (16)	0	0	0	0	1 (33)	0	0	0	0	1 (100)
ML&L	0	10 (53)	1 (5)	0	1 (7)	0	0	0	0	0	0	0
ML	0	0	0	0	0	0	0	0	0	0	0	0
Spirometry n (%)												
Obstructive	65 (57)	16 (84)	16 (89)	13 (93)	11 (79)	6 (86)	2 (67)	2 (100)	2 (100)	1 (100)	1 (100)	0
Non-specific	32 (28)	3 (16)	1 (5)	0	2 (14)	1 (14)	0	0	0	0	0	1 (100)
Normal	3 (3)	0	0	1 (7)	0	0	1 (33)	0	0	0	0	0
Not performed	14 (12)	0	1 (5)	0	1 (7)	0	0	0	0	0	0	0
Microbiology n (%)												
H Influenza	6 (5)	2 (10)	3 (17)	1 (7)	1 (7)	0	0	0	2 (100)	1 (100)	0	0
Mixed population	4 (3)	0	0	0	3 (21)	0	1 (33)	0	0	0	0	0
No pathogen	20 (17)	5 (26)	4 (22)	3 (21)	5 (36)	1 (14)	0	1 (50)	0	0	0	0
Strep pneumo	1 (1)	0	0	0	0	0	0	0	0	0	0	0
Pseudomonas	42 (37)	6 (32)	7 (39)	6 (43)	4 (29)	3 (21)	0	1 (50)	0	0	1 (100)	1 (100)
Staph Aureus	8 (7)	1 (5)	1 (5)	0	0	1 (14)	1 (33)	0	0	0	0	0
B Cepacia	1 (1)	0	1 (5)	0	0	0	0	0	0	0	0	0
Aspergillosis	6 (5)	1 (5)	1 (5)	2 (14)	0	1 (14)	1 (33)	0	0	0	0	0
NTM	2 (2)	0	0	0	0	0	0	0	0	0	0	0
Acinetobacter	4 (3)	0	0	1 (7)	0	0	0	0	0	0	0	0
Complications n (%)												
Pneumonia	4 (3)	3 (16)	0	1 (7)	0	0	1 (33)	0	0	0	0	0
Empyema	0	0	0	0	1 (7)	0	0	0	0	0	0	0
Lung abscess	1 (1)	0	1 (5)	1 (7)	0	0	0	0	0	0	0	0
Respiratory failure	21 (18)	5 (26)	7 (39)	1 (7)	2 (14)	2 (29)	0	0	0	1 (100)	0	0
Cor pulmonale	2 (2)	2 (10)	0	2 (14)	1 (7)	1 (14)	0	0	0	0	0	0
Pneumothorax	0	0	1 (5)	0	0	1 (14)	0	0	0	0	0	1 (100)
Hemoptysis	29 (25)	2 (10)	2 (11)	2 (14)	4 (29)	1 (14)	0	0	1 (50)	0	0	0
Amyloidosis	1 (1)	0	0	0	0	0	0	0	0	0	0	0
None	56 (49)	7 (37)	7 (39)	7 (50)	6 (43)	2 (14)	2 (67)	2 (100	1 (50)	0	1	0
In Hospital outcome n (%)												
DC home with RX	109 (96)	14 (74)	18 (100)	14 (100)	13 (93)	7 (100)	2 (67)	2 (100)	2 (100)	1 (100)	1 (100)	1 (100)
Referred to surgeon	1 (1)	2 (10)	0	0	0	0	1 (33)	0	0	0	0	0
Died	3 (3)	3 (16)	0	0	1 (7)	0	0	0	0	0	0	0

## Discussion

To our best of knowledge, this is the first study from Pakistan on NCFB. Our data showed certain important differences when compared with data published from the rest of the world, however, some findings were similar to previous studies.

Data from the previous studies showed the mean age of patients with NCFB was between 60 and 67 years and the majority were women [3,15,19]. However, in this study majority of patients were men (150, 76.5%) and 163 (83%) of the total patients were younger than 60 years of age.

In terms of clinical signs and symptoms, cough was the most frequent symptom found in 191 (97.4%) patients with daily sputum production in 165 (84.2%) patients which is comparable to the previous data [3,5,9]. This is however interesting to note that despite a large number of the patients had upper lobe bronchiectasis, yet they had complaints of daily sputum production instead of having dry bronchiectasis. There were 82 patients with either UUL or BUL bronchiectasis and out of these 82 patients, 94% (n = 77/82) of patients had post TB bronchiectasis. The productive cough in this group can be explained by the fact that post TB sequelae are just not limited to bronchiectasis, as these patients can also have bronchial distortion, obstructive airway disease unrelated to bronchiectasis, fibrosis, cavitation to name few [20]. For the same reasons despite having localized bronchiectasis, some of these patients had a severe obstruction or nonspecific impairment with very low FEV1 and FVC. So in post TB patients, it is difficult to comment if all the symptoms are due to bronchiectasis only. The majority of the patients in the current study were admitted with an exacerbation of daily symptoms which is likely explained by the inpatient setting of this study.

Among the etiologies, post-infectious causes predominate in this cohort with 58% of patients had a history of pulmonary TB and 9.7% of patients had a history of pneumonia in the past. This is of note that patients with active TB were excluded from the study since HRCT diagnosis of bronchiectasis was a prerequisite for inclusion in the study and in our institute active TB patients do not get HRCT until otherwise indicated for some other indications. The predominance of post TB bronchiectasis is consistent with the high burden of TB in South Asia with Pakistan being ranked 5th among high TB burden countries according to WHO TB report of 2018. However, the proportion of post TB bronchiectasis in our study is even higher than recently published data from the Indian registry of bronchiectasis, i.e., 58.2% vs 35.5% [9]. It can be due to the reason that our institute is one of the largest referral centers for TB patients in Pakistan. The majority of the patients continue to follow up in our institute for their respiratory symptoms even after the cure of TB.

COPD was the 3rd commonest cause of bronchiectasis in this study. Majority of the patients in this group were older than 46 years of age, without evidence of alpha one antitrypsin deficiency and with a smoking history of >15 pack-year. Although COPD is known to start at ages 40 years and onwards, it is important to note that these patients have other risk factors of COPD as well, like Huqqa/Sheesha smoking and significant biomass fuel exposure which cannot be estimated by pack-year of smoking. Biomass fuel smoke, not only associated with COPD but it also causes anthracosis. In previous studies, anthracosis was found to be associated with bronchiectasis as well even in the absence of TB [21]. Although none of the patients in this group underwent bronchoscopy, it is a possibility that these patients have some element of anthracosis as well. So in our population where in rural areas, biomass is still frequently used as fuel for domestic use, it requires separate studies with a large number of patients to see, the incidence of bronchiectasis in this group.

In this cohort, only 7% of the cases remain idiopathic, which is in contrast to the majority of the previous studies where idiopathic cases comprise 18-55% of the study population [3,5,9].

There were 47 patients (24%) who diagnosed to have conditions that altered the management, which is per previous data including COPD, ABPA, hypogammaglobinemia, diffuse pan bronchiolitis, post obstructive etiologies, and CTD. In previous studies, 7-37% cases found to have etiologies which altered the management [12,22].

In terms of radiological pattern, the majority of the cases with post TB bronchiectasis were associated with either unilateral upper lobe or bilateral upper lobe bronchiectasis found in 67.5% (77/114) of patients and nearly 1/4th i.e. 24% (28/114) of patients had diffuse unilateral disease involving all lobes of a hemithorax. The majority of the patients with bronchiectasis secondary to ABPA, COPD, idiopathic, CTD, hypogammaglobinemia, diffuse panbronchiolitis, and PCD had either diffuse bilateral bronchiectasis or bilateral lower lobe bronchiectasis. Two patients in this study developed bronchiectasis with a remote history of foreign body inhalation. In one of the patients, it was removed two years before presentation to our center, however, he already had developed diffuse unilateral bronchiectasis with a history of recurrent exacerbations. The other patient had recurrent lower lobe pneumonia and bronchiectasis for the last five years with no clear history of foreign body inhalation but on bronchoscopy, he was found to have a small plastic piece of probably some toy which was later removed by a thoracic surgeon with rigid bronchoscopy. So bronchoscopy should be considered in evaluation of the cause of localized bronchiectasis.

Like previous studies obstructive impairment was the predominant spirometric pattern in our study (69%) [3,9,23,24]. Due to the unavailability of plethysmograph in our institute, we could not further classify patients with combined low FEV1 and FVC into a restrictive pattern, pseudo restriction because of air trapping or extrapulmonary causes of restriction. It is one of the limitations of this study. It is important that only five patients had normal spirometry. As mentioned earlier, the majority of the patients in our cohort had post TB bronchiectasis, and these patients can have other post TB sequelae leading to abnormal spirometry.

In this study, there is no missing microbiological data as the majority of the patients had a productive cough (n = 165, i.e. 84%) and in the remaining patients, as per study protocol, they underwent bronchoscopy either for localization of hemoptysis or failure to respond on empiric treatment. In terms of microbiology, Pseudomonas was the commonest pathogen isolated which is consistent with some recent data, however, differs from other studies where *Haemophilus influenzae *was the commonest organism among bronchiectasis patients [3,5,9,15,24-26]. The patients in the Pseudomonas group were significantly associated with respiratory failure as compared to non-Pseudomonas group, but interestingly former did not differ significantly with later, in terms of clinical presentation, radiology, spirometry, complications apart from respiratory failure and clinical outcomes. In the majority of the previous studies, Pseudomonas colonization turned out to be the major factor in worse outcomes among bronchiectasis patients [3,9,19,24,26]. It could be partly related to the fact that for a majority of our patients we had no previous data to know if these patients were previously colonized with Pseudomonas or if this was the first-time isolation of Pseudomonas. Similarly, we did not have follow-up data on these patients to know how did they perform after discharge, since a large number of these patients were referred back to their primary physicians in local areas. This we consider one of the weaknesses of our study as we fail to draw definite conclusions in terms of the impact of Pseudomonas in the outcomes of a patient with bronchiectasis.

Hemoptysis was the most frequent complication. In the majority of the patients, it was submassive to massive hemoptysis. A large proportion of these patients responded to antibiotics alone, five of them required bronchial artery embolization and four of them were referred to surgeons for lobectomy. About 1/4th of the patients in this study had a respiratory failure or cor pulmonale. Few patients recovered by the time of discharge and went home without supplemental oxygen but the majority of the patients presenting with these complications required domiciliary oxygen on discharge. This suggests that these patients must have diminished respiratory reserve even before admission to our hospital which in turn points out suboptimal coverage of primary care services in Pakistan as patients seek medical attention very late in the course of disease [27]. This further emphasizes the fact that these patients should be diagnosed and managed earlier to prevent complications as lower respiratory infections are one of the leading causes of death in Pakistan [27]. It becomes even important as so far lung transplant facility is not available in Pakistan, so early diagnosis is the key to prevent death in the younger age group as the majority of the patients in this study were <60 years of age.

During the study, we found rare causes of bronchiectasis as well including diffuse panbronchiolitis, Marfan syndrome, and Mounier-Kuhn syndrome (tracheobronchomegaly). Cases of diffuse panbronchiolitis were diagnosed by the criteria as per the working group of the Ministry of Health and the Welfare of Japan [28]. Mounier-Kuhn syndrome was diagnosed as the diameter of the trachea was 35 mm on CT chest and on bronchoscopy there were diverticulae in airways as described in previous literature [29]. A case of Marfan syndrome with bronchiectasis was diagnosed with typical musculoskeletal features and ectopia lentis as per the Ghent criteria for MFS [30].

There are certain limitations of the study. This is a single-center study where the majority of the patients were referred from rural areas of Sindh and Balochistan, so the results of this study cannot be generalized to the rest of the population of Pakistan. Because of the unavailability of nasal Nitric oxide or electron microscopy, the diagnosis of PCD was made based on dextrocardia, sinusitis, otitis media, infertility, and positive saccharine test. In Pakistan, delta F508 is the only genetic test available for CF so we excluded CF patients by repeating the sweat chloride test on two separate occasions and checking for the only available genetic test for CF. Diagnosis of chronic aspiration just relied on the history and presence of the predisposing conditions which can lead to recurrent aspiration, and as we did not suspect any patient with above, so no further investigations were done. Hence, there is still a possibility that out of the 7% cases of idiopathic bronchiectasis, there may be few with PCD, CF and chronic aspiration. We could get just sputum samples during hospitalization, so isolation of organisms did not confirm if patients were colonized with them. There were patients with previous sputum reports, but since such data was not available for all of them so it was not included.

## Conclusions

In conclusion, our study does point out certain important differences in the etiology, clinical, radiological, and microbiological profile of patients with non-CF bronchiectasis when compared to data from Europe and the USA. Majority of the patients were men, younger than 60 years and post TB bronchiectasis was the leading cause of bronchiectasis. Pseudomonas was the commonest pathogen isolated and hemoptysis was the most frequent complication. As this was a single center study, large multicenter study is needed in order to draw some more generalised conclusions. Diagnostic workup as per BTS guidelines helped in finding the etiologies which lead to change in the patient management.
